# Acidocétose diabétique chez l’enfant: aspects épidémiologiques et pronostiques

**DOI:** 10.11604/pamj.2018.31.167.14415

**Published:** 2018-11-09

**Authors:** Aymar Pierre Gildas Oko, Fayçal Khalil Zaharo Ali, Steve Vassili Missambou Mandilou, Judicaël Kambourou, Lombet Letitia, Jesse Pierre Yolaine Poathy, Moyen Engoba, Mamadou Ildevert Cyriaque Ndjobo, Henri Germain Monabeka, Georges Marius Moyen

**Affiliations:** 1 Faculté des Sciences de la Santé, Université Marien Ngouabi, Brazzaville, République du Congo; 2 Service de Soins Intensifs Pédiatriques, CHU de Brazzaville, République du Congo; 3 Service de Pédiatrie Grands Enfants, CHU de Brazzaville, République du Congo; 4 Service de Pédiatrie Nourrissons, CHU de Brazzaville, République du Congo; 5 Service de Maladies Métaboliques et d’Endocrinologie, BP 32, République du Congo

**Keywords:** Acidocétose, diabète, enfants, décès, facteurs de risque, Brazzaville, Diabetic ketoacidosis, diabetes, children, death, risk factors, Brazzaville

## Abstract

**Introduction:**

Au Congo, les données concernant l'acidocétose diabétique (ACD) chez l'enfant sont anciennes et rares. Notre étude avait pour objectifs de décrire les caractéristiques sociodémographiques de l'ACD et d'identifier les facteurs de risque de décès.

**Méthodes:**

De Janvier 2013 à Juin 2016, nous avons réalisé une étude analytique portant sur l'ACD chez l'enfant au CHU de Brazzaville. Les variables sociodémographiques, cliniques, paracliniques et évolutives ont été étudiées. Les tests de Chi-2, de Fischer et l'odds ratios ont servi pour l'analyse univariée et le modèle de régression logistique pour l'analyse multivariée.

**Résultats:**

Sur 172 enfants hospitalisés pour un diabète, 55(31%) l'étaient pour une acidocétose. Il s'agissait de 33(60%) filles, l'âge moyen: 11,1± 4,9 ans (extrêmes 1 mois et 17 ans), 61,8% des parents avaient un bas niveau socioéconomique. L'acidocétose était révélatrice dans 67,2 % de cas. Le diagnostic avant l'hospitalisation était erroné: 50%. Le facteur déclenchant était souvent infectieux (52,7%). La létalité était de 12,7%. Les facteurs de risque de décès en analyse univariée étaient : l'âge < 5 ans (p=0,000006), le délai de consultation supérieur à 7 jours (p= 0,001), la déshydratation sévère (p = 0,0006), les troubles hémodynamiques (p= 0,0006), la dénutrition sévère (p= 0,02), le Glasgow < 9 (p= 0,007) et la diarrhée (p= 0,001).

**Conclusion:**

L'importance et la gravité de l'acidocétose imposent des mesures de prévention axées sur la sensibilisation, l'information, l'éducation et la maîtrise des facteurs de risque de décès.

## Abstract (en)

**Introduction:**

In Congo, data on diabetic ketoacidosis (DKA) in children are old and rare. This study aims to describe the sociodemographic features of DKA and to identify risk factors for mortality.

**Patients and Methods:**

We conducted an analytical study on DKA in childred at the University Hospital in Brazzaville over the period from January 2013 to June 2016. We examined the socio-demographic, clinical, paraclinical and evolutionary variables. Chi-Square Test, Fisher’s exact test and odds ratio were used in the univariate analysis process and logistic regression model in the multivariate analysis.

**Results:**

Out of 172 children hospitalized with diabetes 55 (31%) were hospitalized with ketoacidosis. They were girls (33; 60%) with an average age of 11.1± 4.9 years (ranging from 1 months to 17 years), 61.8% of parents came from low socioeconomic status. Ketoacidosis was the revealing symptom in 67.2 % of cases. The diagnosis made before hospitalization was wrong (50%). The triggering factor was often an infection (52.7%). Mortality rate was 12.7%. The risk factors for mortality in the univariate analysis were: age < 5 years (p=0,000006), average consultation time higher than 7 days (p= 0.001), severe dehydration (p = 0.0006), hemodynamic disorders (p= 0.0006), severe undernutrition (p= 0.02), Glasgow Coma Scale < 9 (p= 0.007) and diarrhea (p= 0.001).

**Conclusion:**

The importance and the seriousness of ketoacidosis impose preventive measures based on awareness, information and education campaigns as well as on the management of risk factors for mortality.

## Introduction

Le diabète est la première endocrinopathie chez l'enfant. Les données épidémiologiques montrent une constante augmentation de son incidence au niveau mondial. Ce phénomène est attribué à l'augmentation du diabète de type 2 chez les adolescents mais surtout à celle du diabète de type 1, forme la plus observée chez l'enfant [1-3]. L'acidocétose diabétique est la complication aiguë la plus fréquente et la plus redoutée du diabète [4, 5]. Conséquence d'une insulinopénie profonde, l'acidocétose révèle le diabète dans 15 à 70% des cas et, le complique dans 1 à 10% [5, 6]. Elle est la principale cause d'hospitalisation, de morbidité et mortalité des enfants diabétiques en Afrique [7-10]. Son pronostic est fonction de la prise en charge, actuellement bien codifiée et reposant sur la connaissance des facteurs pronostiques ainsi que des mécanismes physiopathologiques [11, 12]. Au Congo, les données concernant l'acidocétose diabétique (ACD) chez l'enfant sont anciennes et rares; l'ACD révèle le diabète dans 57,1% des cas et le complique dans 42,8%, sa létalité est de 18,2% [13]. Dans le but de contribuer à l'amélioration de la prise en charge de l'acidocétose diabétique chez l'enfant, cette étude s'était fixée comme objectifs de décrire les caractéristiques sociodémographiques de l'acidocétose diabétique et d'analyser le pronostic chez l'enfant à Brazzaville.

## Méthodes

**Type et cadre de l'étude**: Il s'est agi d'une étude descriptive et analytique avec recueil des données rétrospectif et prospectif qui s'est déroulée entre Janvier 2013 et Juin 2016 (3 ans et 5 mois), dans les services de soins intensifs pédiatriques (SIP) et de maladies métaboliques et endocriniennes (MME) du Centre Hospitalier et Universitaire de Brazzaville (CHU-B). Ces deux services accueillent la quasi-totalité des enfants diabétiques de la ville de Brazzaville.

**Critères d'inclusion**: Les enfants âgés d'un mois à dix-sept ans hospitalisés dans les services sus-cités, répondant aux critères diagnostiques d'ACD définis pour l'étude ont été inclus de façon systématique et consécutive. Le consentement éclairé des parents était exigé. Les variables d'étude étaient d'ordre :

**Sociodémographique**: L'âge, le sexe et la provenance des enfants, le niveau socioéconomique (évalué selon la classification de Gayral-Taminh) [14] et le niveau d'instruction des parents. les antécédents familiaux et personnels de diabète.

**Clinique**: Les circonstances du diagnostic, le diagnostic retenu avant l'hospitalisation, le temps écoulé entre le début des symptômes et l'hospitalisation; le motif de consultation, le facteur déclenchant l'acidocétose, les signes de l'examen physique: l'état de conscience (évaluée selon le score de Glasgow [15, 16]),l'état nutritionnel (évalué selon les critères de l'OMS [17]),l'état d'hydratation (normal, déshydratation modérée, déshydratation sévère), l'état hémodynamique, la dyspnée de Küssmaul, l'odeur acétonique de l'haleine.

**Paraclinique**: La glycémie (la glycémie capillaire mesurée avec l'appareil AccuChek performa^®^ et veineuse si besoin), la glycosurie et la cétonurie dosées à la bandelette urinaire réactive, l'ionogramme plasmatique, la numération formule sanguine (NFS), la protéine C réactive (CRP). Les autres examens étaient demandés en fonction du contexte clinique.

**Thérapeutique**: La quantité d'apport liquidien journalier. La dose quotidienne de l'insuline.

**Evolutive**: L'évolution de la glycémie, le délai de la négativation de la cétonurie, les complications (hypoglycémie, œdème cérébral), la sortie, le décès, la durée d'hospitalisation.

### Déroulement de l'étude

Pour la réalisation de l'étude, une fiche d'enquête préétablie a été élaborée et remplie par le même examinateur. Les parents et les enfants en âge de s'exprimer ont été interviewés. Les médecins traitants et les dossiers médicaux ont également constitué nos sources de données.

### Définitions opérationnelles

**L'acidocétose diabétique**: Classiquement, le diagnostic d'ACD est retenu sur des critères biologiques : glycémie ≥2g/L (11 mmol/L), glycosurie ≥2 croix, cétonurie ≥2 croix, pH < 7,3 ou réserve alcaline < 15 mmol/L [11]. Ne pouvant pas réaliser la gazométrie (pH, réserve alcaline) dans la présente étude, le diagnostic d'ACD a été retenu devant l'association d'au moins un des signes cliniques suivants : vomissements, douleurs abdominales, dyspnée de Küssmaul, signes de déshydratation et trouble de la conscience aux signes biologiques suivants : glycémie ≥2g/L (11mmol/L), glycosurie ≥ 2 croix, cétonurie ≥2 croix.

**L'hypoglycémie**: était définie comme une glycémie < 0,7g/L (3,3mmol/L); elle était dite sévère lorsque la glycémie était ≤0,40g/L (2,2mmol/L) [11].

**L'œdème cérébral**: En l'absence d'imagerie cérébrale, le diagnostic d'œdème cérébral était évoqué devant:

**Les critères majeurs**: Une altération de la conscience, une bradycardie.

**Les critères mineurs**: Les céphalées, nausées, vomissements, HTA avec PAD > 90 mmHg, âge < 5 ans. Ce diagnostic était suspecté devant l'existence de deux critères majeurs ou l'association d'un critère majeur à deux critères mineurs. La sensibilité de ces critères diagnostiques est de 92% [18].

### Analyse statistique

Le traitement et l'analyse des données ont été réalisés au moyen du logiciel Epi Info version 7.2.0.1. les variables quantitatives ont été exprimées en moyenne ± écart type lorsque la distribution était normale ou en médiane et intervalle interquartile (IQR) au cas contraire. Les variables qualitatives ont été exprimées en pourcentage. Les effectifs de chaque variable ont également été précisés. L'analyse univariée a été réalisée avec le test Chi2 de Pearson ou le test exact de Fisher et le odds ratios (OR) pour les variables catégorielles. L'analyse multivariée a été réalisée à l'aide du modèle de régression logistique. Le seuil de significativité était inferieur 5% et intervalle de confiance à 95%.

### Considérations éthiques

Pour chaque enfant le consentement éclairé des parents ou des tuteurs a été obtenu. L'étude a été réalisée dans le respect de la déclaration d'Helsinki [19]. L'étude a été approuvée par le comité national d'éthique en sciences de la santé sous le numéro 1029.

## Résultats

### Aspects sociodémographiques et cliniques

**Figure 1:**
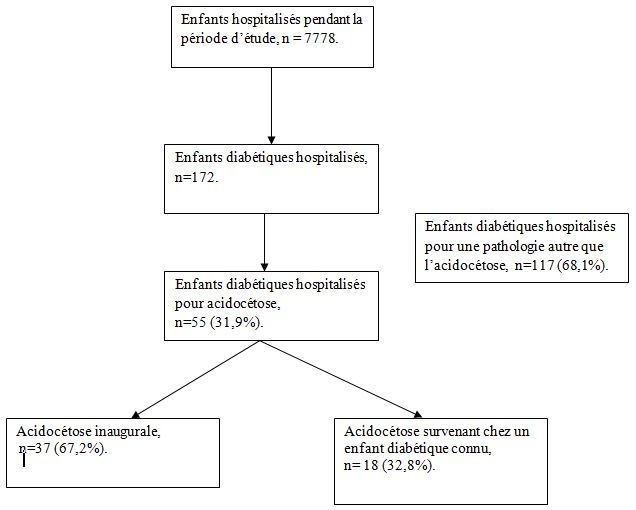
Diagramme de flux de sélection de la population d’étude

Durant la période d'étude, 7778 enfants étaient hospitalisés dans les services de soins intensifs pédiatriques et de maladies métaboliques et endocriniennes. Parmi eux, 55 étaient hospitalisés pour une ACD, soit une fréquence globale de 0,7% (Figure 1). La répartition annuelle de l'incidence se présentait ainsi qu'il suit : 18,8% en 2013, 29,5% en 2014 et 40% en 2015. Il s'agissait de 33 filles (60%) et l'âge moyen était de 11,1 ± 4,9 ans (extrêmes 1 mois et 17 ans). Les parents avaient majoritairement un niveau socioéconomique bas n= 34 (61,8%). Trente-deux enfants (58,2%) venaient directement du domicile et 23 (41,8%) étaient référés par une structure sanitaire. Une consultation médicale était faite dans un centre de santé avant l'hospitalisation pour 28 enfants (50,9%). Le diagnostic retenu était incorrect pour 14/28 enfants (50%), et 13 (92,9%) de ces 14 enfants n'étaient pas connus diabétique. Le délai médian de recours à l'hôpital était de 5,7 jours (IQR, 4,6-10,2 jours). Les caractéristiques sociodémographiques et cliniques sont détaillées dans le Tableau 1.

**Tableau 1:** Caractéristiques sociodémographiques, cliniques des enfants hospitalisés pour acidocétose diabétique

Caractéristiques	N (%)	Fréquence cumulée (%)
**Tranche d’âge (an)**		
[0-5[	8(14,5)	14,5
[5-10[	11 (20,0)	34,5
[10-17]	36(65,5)	100,0
**Sexe**		
Féminin	33 (60,0)	60,0
Masculin	22 (40,0)	100
**Niveau socioéconomique des foyers**		
Elevé	8(14,6)	14,6
Moyen	13 (23,6)	38,2
Bas	34(61,8)	100,0
**Niveau d’instruction des parents**		
**Mère**		
Primaire	25 (45,4)	45,4
Secondaire	27 (49,1)	94,5
Universitaire	3 (5,5)	100,0
**Père**		
Primaire	4 (7,3)	7,3
Secondaire	19 (34,5)	41,8
Universitaire	32 (58,2)	100,0
**Antécédents de diabète**		
Aucun	33 (60,0)	60,0
Premier degré	7 (12,7)	72,7
Deuxième degré	13 (23,7)	96,4
Autres liens	2 (3,6)	100,0
**Délai de recours à l’hôpital (jours)**		
[0-3[	4 (7,2)	7,2
[3-7[	31 (56,4)	63,6
≥ 7	20 (36,4)	100,0
**Score de Glasgow**		
15	27 (49,1)	49,1
9-14	4 (7,3)	56,4
<9	24 (43,6)	100,0
**Troubles hémodynamiques**		
Oui	21 (38,2)	38,2
non	34 (61,8)	100,0
**Etat d’hydratation**		
Normal	1 (1,8)	1,8
Déshydratation modérée	33 (60)	61,8
Déshydratation sévère	21 (38,2)	100,0
**Etat nutritionnel**		
Normal	25 (45,5)	45,5
Dénutrition modérée	16 (29,1)	74,6
Dénutrition sévère	11 (20,0)	94,6
Surpoids	0 (0,0)	94,6
Obèse	2 (3,6)	100,0
**Température**		
Normothermie	8(14,5)	14,5
Hypothermie	41 (74,5)	89,0
Hyperthermie	6 (11,0)	100,0
**Dyspnée**		
Oui	18 (32,7)	32,7
non	37 (67,3)	100,0

**Figure 2:**
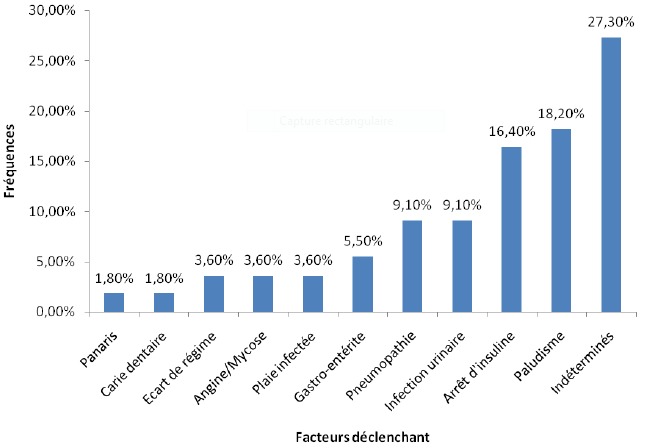
Facteurs favorisant la décompensation du diabète chez les enfants hospitalisés au CHU de Brazzaville pour acidocétose diabétique

**Facteurs déclenchant l'acidocétose**: Le facteur responsable de la décompensation du diabète était retrouvé chez 40 enfants (72,7%): Figure 2.

**Signes paracliniques**: Le taux médian de la glycémie à l'admission était de 26,3mmol/L (IQR, 22,0-28,8 mmol/L), (extrêmes17,1 et 35,8mmol/l). La cétonurie était à 2+ pour 3enfants (5,5%), à 3+ pour 15 enfants (27,3%) et à 4+ pour 37 enfants (67,3%). La glycosurie était à 2+ pour 2 enfants (3,6%), à 3+ pour 13 enfants (23,6%) et à 4+ pour 40 enfants (72,7%). L'ionogramme plasmatique réalisé chez 15(27, 3%) enfants après au moins 24 heures de traitement montrait : une natrémie normale dans tous les cas, une kaliémie normale chez 12 enfants et basse chez les 3 restants, respectivement à 3,3 mmol/L, 3,2 mmol/L et 3,0 mmol/L. La créatininémie réalisée en même temps que l'ionogramme plasmatique était normal chez 12 enfants et élevée chez 3 enfants avec les valeurs respectives de 17 mg/L, 20 mg/L et 25 mg/L.

### Aspects thérapeutiques

**La réhydratation**: Au cours des premières 24h, la quantité moyenne de soluté administrée était de 2215,4 ± 995,6mL/m^2^, (extrêmes 250 et 4800mL/m^2^).

**L'insulinothérapie**: La dose moyenne d'insuline administrée au cours des premières 24 h était de 9,90 UI±3,94 (extrêmes 4,80 et 14,40) chez les enfants de moins de 60 mois et de 76 UI ± 28,06 (extrêmes 36 et 132) chez ceux âgés d'au moins 60 mois.

**Aspects évolutifs**: La durée moyenne d'hospitalisation était de 8,7 ± 4 jours, (extrêmes 2 et 17 jours). L'évolution était favorable pour 48 enfants (87,2%). une complication était observée chez 17 (30,8%) enfants. Il s'agissait d'un œdème cérébral n = 2 (3,6%) et une hypoglycémie sévère n = 15 (27,2%). L'évolution s'est faite vers un décès chez 7 enfants (12,7%). Il s'agissait de 5 garçons et deux filles âgés en moyenne de 2,8 ans (extrêmes de 1mois et 15 ans). Le décès était survenu au cours des 24 premières heures d'hospitalisation dans tous les cas. Les facteurs associés au décès en analyse univariée figurent au Tableau 2. Après ajustement, aucune variable n'était associée au risque accru de décès. Les causes de décès étaient le choc (n=4), l'œdème cérébral (n=2) et l'hypoglycémie sévère (n=1).

**Tableau 2:** Facteurs associés au décès chez les enfants hospitalisés pour ACD diabétique

Variables	Décédés	Analyse univariée		Analyse multivariée	
	N (%)	P-value	OR [IC à 95%]	*p*-value	OR [IC à 95%]
Age < 5 ans	6 (75,0)	0,000006*	138,0 [10,8-1761,7]	0,05	55,45 [0,98- 3122,87]
Délai d’admission > 7j	6(37,5)	0,001*	22,8 [2,5- 211,8]	0,29	6.42 [0,20- 199,74]
dénutrition sévère	4(36,4)	0,02*	7,8 [1,4- 42,7]	0,93	1,18 [0,02- 54,13]
Déshydratation sévère	7(33,3)	0,0006*	Non défini	0,97	-
Troubles hémodynamiques	7(33,3)	0,0006*	Non défini	0,97	-
Glasgow < 9	6(30,0)	0,007*	14,6 [1,6- 132,3]	0,24	6,76 [0,29- 160,26]
Diarrhée	4(66,7)	0,001*	30,6 [3,9- 240,7]	0,44	4,88 [0,08 – 282,80]
Fièvre	4(25,0)	0,17	4,0 [0,8 - 20.5]	0,95	1,10 [0,03- 35,06]

## Discussion

La présente étude consacrée à l'acidocétose diabétique de l'enfant à Brazzaville, a connu un certain nombre d'écueils. Le premier concernait la faible taille de la population d'étude, qui a limité la puissance des tests de comparaison utilisés et n'a pas permis d'identifier les facteurs de risque de décès en analyse multivariée. Cette difficulté est inhérente aux études réalisées sur les pathologies rares comme l'est l'acidocétose diabétique chez l'enfant à Brazzaville. La réalisation d'une étude sur une période beaucoup plus longue était souhaitable pour avoir un échantillon beaucoup plus important. Par contre les lieux et le mode de recrutement de la population d'étude rendent celle-ci représentative de la population d'enfants hospitalisés pour acidocétose diabétique à Brazzaville. Enfin, l'insuffisance du plateau technique, n'a pas permis la réalisation en temps voulu de certains examens complémentaires comme l'ionogramme sanguin, créatininémie et l'ECG. D'autres examens importants pour le diagnostic: le pH sanguin, la réserve alcaline, l'IRM cérébrale n'ont pas été réalisés justifiant l'élaboration de critères diagnostiques opérationnels. Au Congo, les données concernant l'acidocétose diabétique chez l'enfant sont rares et anciennes [13, 20]. La présente étude a montré que l'acidocétose diabétique occupait une place mineure dans la pathologie de l'enfant à Brazzaville, mais était par son incidence l'une des principales causes d'hospitalisation de l'enfant diabétique. L'incidence de l'acidocétose diabétique dans cette étude était identique à celle rapportée par d'autres auteurs [21-25], mais supérieure à celle observée dans certains pays de l'Europe [26-28]. A Brazzaville, la population d'enfants hospitalisés pour acidocétose diabétique est en augmentation régulière; en effet, l'incidence annuelle de celle-ci a plus que doublée durant la période de l'étude, passant de 18,6 à 40% entre 2013 et 2015. De même, le nombre d'enfants hospitalisés par année pour acidocétose diabétique dans ce travail était plus important que celui rapporté dans les travaux de Monabeka *et al*. réalisés dans les mêmes services [13, 20] (55 cas sur 3,5 ans Vs 77 sur 9 ans).

L'augmentation de l'incidence de l'acidocétose diabétique était corrélée à celle de l'acidocétose diabétique révélatrice: 67,2% de la population de notre étude vs 57,1% dans l'étude de Monabeka [20]. L'augmentation de l'incidence de l'acidocétose diabétique révélatrice, ainsi que le taux élevé de diagnostics erronés et tardifs observés dans la présente étude, laissent penser à une méconnaissance du diabète de l'enfant et de ses complications par la population et les professionnels de santé. Robert en France, avait noté que 28% des médecins généralistes ne savaient pas que le diabète existe avant l'âge de 2 ans [29]. Les auteurs africains consultés avaient fait le même constat [8, 30]. Un travail de sensibilisation, d'information, d'éducation devrait être mené auprès des professionnels santé et de la population générale pour espérer baisser comme dans certains pays développés l'incidence de l'acidocétose diabétique [31-33]. A contrario, la faible représentation d'enfants diabétiques connus était la preuve du bon suivi de ceux-ci grâce entre autres à un meilleur accès au lecteur de glycémie capillaire et à l'insuline rendus gratuits depuis quelques années. L'accroissement de la population d'enfants hospitalisés pour acidocétose diabétique s'accompagnait d'un rajeunissement de celle-ci, en effet 14,5% de notre population d'étude avait au plus 4 ans, à la différence des études précédentes, où tous les enfants avaient plus de 4 ans [13, 20]. Ce rajeunissement déjà constaté dans les pays développés [34-36] risque de s'accentuer en Afrique puisque l'augmentation du diabète chez les tout-petits s'accélère [1, 37]. Outre le fait que la population d'enfants diabétiques devienne de plus en plus jeune, le rajeunissement de la population d'enfants hospitalisés pour acidocétose peut être également expliqué par la difficulté d'identifier les premiers signes du diabète et la présentation clinique en acidocétose plus fréquente suite à une plus rapide destruction des cellules bêta de Langerhans pancréatiques chez le jeune enfant [36-38].

Malgré le rajeunissement de notre population d'étude, l'acidocétose diabétique demeure en Afrique, une pathologie de l'adolescent, puisque les enfants âgés de 10 ans et plus (10 -17 ans) étaient dans ce travail comme dans ceux des autres auteurs africains les plus touchés [8, 10, 13]. Le lien entre le niveau social et l'acidocétose diabétique est connu, parce que déjà rapporté [10, 36, 39, 40]. Dans notre série, les enfants dont les parents avaient un bas niveau socio-économique étaient les plus concernés. Le faible revenu familial et l'absence de couverture sociale rendent l'accès aux soins difficiles majorant ainsi le risque d'acidocétose [36, 40]. Les facteurs déclenchant l'acidocétose diabétique sont les mêmes partout. Ils sont dominés par les infections au premier rang desquelles le paludisme en Afrique sub-saharienne, et l'inobservance de l'insulinothérapie chez l'enfant diabétique connu [8, 10, 24, 41, 42]. L'abandon du traitement, principale cause de l'acidocétose diabétique dans l'étude de Monabeka [13], était retrouvé dans une proportion moindre dans cette étude, preuve d'un meilleur accès à l'insuline et d'une meilleure éducation thérapeutique des patients. L'acidocétose diabétique, bien que rare chez l'enfant, était responsable de 12,7 % des décès. Cette létalité, bien qu'en net recul [13], reste élevée. La maîtrise de la létalité nécessite que soit connus les facteurs de risque de décès. Lesquels facteurs identifiés dans cette étude uniquement en analyse univariée (l'âge < 5 ans, le diagnostic tardif au-delà de 7 jours, l'existence des troubles hémodynamiques, d'une déshydratation sévère, d'une dénutrition sévère et d'un score de Glasgow < 9) en raison d'une faible taille de la population d'étude, devraient être confirmés en analyse multivariée dans une étude avec une population de taille suffisante. Les causes de décès dans cette étude sont identiques à celles retrouvées par d'autres auteurs [10, 43, 44].

## Conclusion

L'acidocétose diabétique est une pathologie peu fréquente mais en augmentation chez l'enfant à Brazzaville. Elle demeure grave en raison de la forte létalité. Les facteurs de risque de décès identifiés en analyse univariée: l'âge < 5ans le diagnostic tardif au-delà de 7 jours, l'existence des troubles hémodynamiques, d'une déshydratation sévère, d'une dénutrition sévère, d?un score de Glasgow < 9 et la diarrhée, devraient être confirmés en analyse multivariée dans une étude avec une population de taille suffisante. Des mesures de lutte efficaces doivent être mises en œuvre, pour éviter que l'acidocétose diabétique devienne un véritable problème de santé publique chez l'enfant.

### Etat des connaissances actuelles sur le sujet

- L'incidence du diabète est en augmentation chez l'enfant au niveau mondial;
- L'acidocétose diabétique est la complication aiguë la plus fréquente et la première cause de décès chez l'enfant;
- La prévalence et le pronostic de l'acidocétose diabétique varient d'un pays à un autre en fonction aussi bien de la connaissance qu'ont les populations et les professionnels de santé du diabète que de la qualité de la prise en charge du diabète et de l'acidocétose.

### Contribution de notre étude à la connaissance

- Notre étude est la deuxième consacrée spécifiquement à acidocétose diabétique chez l'enfant au Congo. On observe une augmentation et un rajeunissement de la population d'enfants hospitalisés pour acidocétose diabétique;
- La mortalité liée à l'acidocétose diabétique demeure importante chez l'enfant;
- Nous avons identifié les principaux facteurs de risque de décès.

## Conflits d’intérêts

Les auteurs ne déclarent aucun conflit d'intérêts.
